# Short-term high-intensity resistance training: a feasibility study on pulmonary, immune and physical-functional fitness benefits for older adults with metabolic syndrome

**DOI:** 10.1007/s00421-025-05920-0

**Published:** 2025-07-26

**Authors:** Juliana de Melo Batista dos Santos, Guilherme Eustáquio Furtado, Eviton Correa-Sousa, Maysa Alves Rodrigues Brandao-Rangel, Manoel Carneiro Oliveira-Junior, Katielle Rodrigues da Silva Cardoso, Mariana Alvarez de Souza, Francisco Rodrigues, Patricia Coelho, Luís Vicente Franco de Oliveira, André Luís Lacerda Bachi, Luciana Malosa Sampaio Jorge, Patrícia Sardinha Leonardo Lopes-Martins, Regiane Albertini, Rodolfo P. Vieira

**Affiliations:** 1 Post-Graduate Program in Sciences of Human Movement and Rehabilitation, Federal University of São Paulo (UNIFESP), Santos, SP Brazil; 2 Polytechnic University of Coimbra, Rua da Misericórdia, Lagar dos Cortiços – S. Martinho do Bispo, 3045-093 Coimbra, Portugal; 3 SPRINT - Sport Physical Activity and Health Research & Innovation Center, Polytechnic University of Coimbra, Rua Dom Joao III – Solum, 3030-329 Coimbra, Portugal; 4 Center for Studies on Natural Resources, Environment, and Society (CERNAS), Polytechnic University of Coimbra, Bencanta, 3045-601 Coimbra, Portugal; 5 Laboratory of Pulmonary and Exercise Immunology (LABPEI), Evangelical University of Goiás (Unievangélica), Anápolis, GO Brazil; 6 Polytechnic University of Castelo Branco, Castelo Branco, Portugal; 7 Post-Graduation Program in Health Science, Santo Amaro University (UNISA), São Paulo, SP Brazil; 8 Nove de Julho University, São Paulo, SP Brazil

**Keywords:** Aging, Immune system, Lung mechanics, Metabolic syndrome, Resistance training

## Abstract

The incidence of metabolic syndrome (MetS) is rising rapidly, particularly among older adults, and is associated with comorbidities that impair respiratory and immune functions. Physical exercise has proven effective in mitigating the adverse effects of both aging and MetS. However, evidence on the impact of high-intensity resistance training (HIRT) on the respiratory and immune systems in older adults with MetS remains limited. This study aimed to evaluate the effects of HIRT on respiratory function, skeletal muscle strength, and immune modulation in older adults with MetS, highlighting its potential as a complementary therapeutic approach. A total of 43 older adults with MetS were enrolled and divided into two groups: a HIRT intervention group (n = 23; mean age 66.71 ± 4.98 years) and a non-exercising control group (n = 20; mean age 66.91 ± 5.26 years). The HIRT protocol involved twice-weekly sessions (10 total) over 5 weeks, performed at 80–90% of one-repetition maximum. Results showed that HIRT significantly improved lung mechanics (R5Hz, R20Hz, Z5Hz, X5Hz), peripheral muscle strength, and both maximal expiratory and inspiratory pressures. Furthermore, HIRT increased anti-inflammatory and anti-fibrotic cytokines in sputum (klotho, IL-10, adiponectin) and serum (klotho, relaxin-1, relaxin-3, IL-10), while reducing pro-inflammatory cytokines in sputum (IL-6, TNF-α) and serum (IL-1ra, IL-6, TNF-α, leptin). A decrease in total leukocyte, neutrophil, lymphocyte, and monocyte counts was also observed. In conclusion, HIRT effectively mitigates the effects of MetS on respiratory, muscular, and immune functions in older adults and may be recommended as a complementary strategy for managing MetS in this population.

**Supplementary Information:**

The online version contains supplementary material available at 10.1007/s00421-025-05920-0.

## Introduction

Aging is a physiological process that affects all organs and systems, including the respiratory and immune systems (Brandsma et al. 2017). The decline in lung function associated with aging is attributed to structural changes in the lungs, alterations in the chest wall, and weakening of the respiratory muscles (Cho and Stout-Delgado 2019). These age-related changes predispose individuals to several respiratory diseases, including chronic obstructive pulmonary disease (COPD) (Buist et al. 2007). Similarly, the aging immune system undergoes “immune senescence,” characterized by a dysregulated immune response that reduces its ability to effectively combat infections and inflammation (Montgomery and Shaw 2015; Witkowski 2022).

Metabolic syndrome (MetS) is defined by the simultaneous presence of at least three of the following factors: insulin resistance, hyperinsulinemia, glucose intolerance, type 2 diabetes, systemic hypertension, central obesity, and dyslipidemia (Zafar et al. 2018). Among these factors, obesity is particularly associated with reduced lung function (Dixon and Peters 2018), impaired pulmonary mechanics, and a weakened immune response (Andersen et al. 2016).

Despite the negative impacts of MetS, epidemiological studies consistently highlight the benefits of regular physical activity in both preventing and managing the condition (Chomiuk et al. 2024). The effects of aerobic training on reducing blood pressure and increasing HDL-c in individuals with MetS are well established (Lemes et al. 2018). In particular, resistance training (RT) is recommended to counteract muscle mass loss (Roie et al. 2013), improve strength, and mitigate the age-related decline in immune function (Cao Dinh et al. 2019). Recently, time-efficient exercise protocols, such as high-intensity resistance training (HIRT), have emerged as effective alternatives to traditional, prolonged exercise regimens (Moro et al. 2017). These intermittent protocols offer older adults with MetS a practical and efficient way to achieve significant health benefits without requiring a substantial time commitment (Jones et al. 2024).

High-intensity resistance training (HIRT), characterized by resistance exercises performed at 80–90% of one-repetition maximum, has gained attention as an effective strategy to address both the physiological and metabolic challenges associated with aging and MetS (Islam et al. 2022; Jones et al. 2024). Its simplicity and time efficiency make it particularly suitable for older adults who may have difficulty adhering to conventional exercise regimens (O’Brien et al. 2020). Several studies have shown that even brief bouts of resistance training can lead to significant improvements in strength, muscle mass, and metabolic function (Shojaa et al. 2020; Lopez et al. 2017). However, although the benefits of HIRT in younger populations are well established (O’Brien et al. 2020), its effects on respiratory health and immune function in older adults with MetS remain poorly understood.

Recent evidence suggests that HIRT may improve pulmonary mechanics and strengthen respiratory muscles, which are often compromised in individuals with MetS (Craighead et al. 2021). Moreover, the immunomodulatory effects of exercise are also noteworthy, as regular exercise has been shown to enhance immune responses, reduce systemic inflammation, and increase levels of beneficial anti-inflammatory cytokines (Lemes et al. 2018; Syed et al. 2024). These changes could potentially counteract the immune dysfunction associated with MetS and aging.

Despite these promising findings, the feasibility and effectiveness of HIRT in improving both pulmonary and immune responses in older adults with MetS have not been thoroughly explored. Given the increasing prevalence of MetS and the challenges associated with conventional exercise protocols, the development of time-efficient, targeted interventions is crucial. The present study aims to bridge this gap by investigating whether a short-term HIRT protocol can improve pulmonary function, immune response, and overall health outcomes in older adults diagnosed with MetS. We hypothesize that such an approach could offer significant health benefits for older adults facing the challenges of MetS.

## Materials and methods

### Study design

This was a two-arm interventional feasibility study conducted over a 5-week period to evaluate the effects of HIRT on pulmonary, muscular, and immune parameters in older adults with metabolic syndrome (MetS). The study adhered to established guidelines to ensure transparency, quality, and reliability in the reporting of pilot and feasibility studies (Lancaster and Thabane 2019).

### Participants and settings

The study involved older adults recruited from São José dos Campos, São Paulo, Brazil, who were allocated into two groups: a HIRT intervention group and a non-exercising control group (CG). All participants attended sessions at the Laboratory of Pulmonary Immunology and Exercise (LABPEI) at the Federal University of São Paulo (UNIFESP), a facility equipped for HIRT, pulmonary function assessments, and biochemical sample collection. Training and assessments were supervised by qualified exercise physiologists and healthcare professionals to ensure participant safety and compliance with the study protocol.

### Sample size calculation

Based on the methodology of previous studies in similar populations, such as the study by Moro et al. (2017), which examined HIIRT in older adults, an effect size (Cohen's d) of 0.80 was assumed for the primary outcomes (e.g., improvements in strength and body composition). A power of 0.80 and a significance level of 0.05 were used for the calculation (Faul et al. 2007). Based on these assumptions, the minimum required sample size for each group was calculated to be 20 participants, ensuring sufficient power to detect meaningful differences in the effects of high-intensity resistance training on pulmonary, muscular, and immune parameters.

### Selection criteria of participants

Initially, thirty older adults diagnosed with metabolic syndrome were selected for participation. To be eligible for the study, participants had to meet the following criteria: (i) be aged 60 years or older; (ii) have a clinical diagnosis of metabolic syndrome; and (iii) have no decompensated respiratory, cardiovascular, muscular, or skeletal diseases. Participants were excluded if they: (i) did not complete at least 90% of the exercise sessions; or (ii) did not participate in all the assessments. After applying the inclusion and exclusion criteria, twenty-three participants were included in the study. All twenty-three participants completed the short-term HIRT protocol and all pre- and post-intervention assessments.

### Ethical issues

The study was conducted in accordance with Resolution 196/1996 of the Brazilian National Health Council (Novoa 2014) and followed the guidelines for ethics in scientific experiments in exercise science research (Shephard 2002). It also complied with the principles outlined in the Declaration of Helsinki for research involving human subjects (Review et al. 2014). All participants signed an informed consent form prior to enrollment, confirming voluntary participation and understanding of the study’s purpose, procedures, and potential risks.

### Exercise protocol

The HIRT protocol was developed based on established recommendations for RT in older adults (Zaleski et al. 2016), including guidelines from the American College of Sports Medicine (ACSM) and the National Strength and Conditioning Association (NSCA) (Garber et al. 2011; Fragala et al. 2019). These guidelines emphasize the importance of intensity, progression, and exercise variety to enhance muscular strength and functional capacity while minimizing injury risk in older populations. HIRT sessions were conducted twice a week over the course of five weeks. The regimen adhered to these recommendations to ensure both safety and effectiveness, particularly for individuals with MetS.

The exercises performed included bent-over barbell row, deadlift, flat bench press, and leg press at a 45° angle, each executed at an intensity of 80–90% of one-repetition maximum (1RM) (Fragala et al. 2019). Each session consisted of four sets of 4–8 repetitions, with rest intervals of 1 min and 30 s to 2 min between sets. The first session served as a familiarization phase, allowing participants to learn the correct techniques and ensure safe performance throughout the protocol (see Table S1 in the supplementary material).

## Measurements

### Anthropometric and body composition analysis

Body weight and height were measured using a standardized scale with an integrated stadiometer, and the body mass index (BMI) was calculated (Lohman et al. 1992). Waist circumference was measured at the narrowest point between the last rib and the iliac crest, and hip circumference was measured at the widest part of the buttocks. These measurements were used to calculate the waist-to-hip ratio (WHR), a critical indicator of central adiposity and associated health risks. Body composition was evaluated using a multifrequency octopolar bioimpedance device [Bioscan 920-II-S, Maltron Inc., UK] (Kafri et al. 2013).

### Health-related quality of life (HrQoL) assessment

The Short Form-36 (SF-36) questionnaire was used to assess HrQoL, encompassing eight domains: functional capacity, physical limitations, pain, general health status, vitality, social aspects, emotional limitations, and mental health. Each domain is scored on a scale from 0 to 100, where 0 represents the worst health status and 100 indicates the best possible health status (Laguardia et al. 2013).

### Lung function (spirometry) and mechanics analysis

Lung function was measured using the Master Screen spirometer (Jaeger, Germany) with forced maneuvers, adhering to American Thoracic Society standards (Berntsen et al. 2016). Parameters analyzed included forced vital capacity (FVC), forced expiratory volume in one second (FEV_1_), and the Tiffeneau index (FEV_1_/FVC). Results were expressed as percentages of predicted values for the Brazilian population.

Lung mechanics were evaluated using the Impulse Oscillometry System (IOS) with the Master Screen oscillometer (Jaeger, Germany) (Berger et al. 2021). Parameters included total respiratory system resistance (R5Hz), proximal airway resistance (R20Hz), distal airway resistance (R5Hz-R20Hz), respiratory system impedance (Z5Hz), and reactance (X5Hz). Results were expressed as percentages of predicted values or absolute values.

### Physical-functional fitness variables (PFF)

The PFF variables assessed in this study encompass key components of functional and respiratory performance, providing a comprehensive evaluation of participants' overall physical capacity. These variables included: (a) maximal inspiratory pressure (MIP) and maximal expiratory pressure (MEP), measured using an analog manovacuometer following international guidelines (Rodrigues et al. 2017), with results presented in absolute values (cm H2O); (b) flexibility, assessed using the Sit-and-Reach test (SRT), which evaluates the ability to stretch the lower back and hamstring muscles; (c) aerobic capacity, assessed through the 6-Minute Walking Test (6MWT), measuring the total distance covered in six minutes as an indicator of endurance and cardiovascular fitness; (d) anaerobic capacity and muscle resistance strength were evaluated via the 30-Second Sit-to-Stand Test (30 s-SS), which determines the number of sit-to-stand repetitions completed in 30 s, reflecting lower limb strength and endurance; (e) the stationary march test (SMT) which measure coordination, balance, and lower body endurance by evaluating participants' ability to perform stationary marching (Langhammer and Stanghelle 2015); (f) handgrip strength (HGS), measured using a hand dynamometer to determine maximal isometric strength in both the left and right hands (Amaral et al. 2019).

### Induced sputum collection and analysis

Sputum samples were collected following inhalation of hypertonic saline (3% NaCl) after administration of 400 μg of salbutamol sulfate (Ten Brinke et al. 2001). Samples were processed with dithiothreitol to separate mucus and cells, and cytological evaluation was performed using Diff-Quik staining (Goncalves and Eze 2023).

### Breath condensate collection and analysis

Exhaled breath condensate (BC) was collected using the RT-Tube (Respiratory Research, USA) over a period of 15–20 min. Samples were stored at −86 °C until analysis of pro-inflammatory, anti-inflammatory, pro-fibrotic, and anti-fibrotic mediators (Rodriguez et al. 2023).

### Blood collection and analysis

Blood samples (5 mL) were collected, and 25 μL was analyzed using an automated hematocytometer (Sysmex XS-800i, Sysmex Europe GmbH, Germany). Serum was separated by centrifugation and stored at −86 °C for further analysis (Fujimaki et al. 2024).

### Cytokine and growth factor quantification

Cytokines and growth factors in serum and breath condensate (BC) were measured using ELISA DuoSet kits (R&D Systems, USA), as described by previous study (Geladaris et al. 2023). The following were analyzed: pro-inflammatory cytokines [Interleukin-1 beta (IL-1β), Interleukin-6 (IL-6), Interleukin-1 receptor antagonist (IL-1ra), Interleukin-8 (IL-8), Tumor Necrosis Factor-alpha (TNF-α)], anti-inflammatory cytokine [Interleukin-10 (IL-10)], pro-fibrotic marker [Vascular Endothelial Growth Factor (VEGF)], and anti-fibrotic markers (Relaxin-1, Relaxin-3, Klotho). Results were expressed in picograms per milliliter (pg/mL).

### Statistical analysis

Statistical analyses and graph generation were performed using GraphPad Prism 5.0 software. Sample size estimation was conducted using G*Power software to ensure adequate statistical power for the study design (Faul et al. 2007). To compare baseline differences between groups, Cohen's *d* effect size was calculated, with thresholds for small (0.2), medium (0.5), and large (0.8) effects (Hopkins et al. 2009). For within-group comparisons, a paired *t*-test was employed, with significance set at *p* < 0.05. Results are expressed as mean ± standard deviation. Between-group comparisons were conducted only at baseline to confirm the similarity between groups prior to the intervention. Since the control group did not exhibit significant changes over time, the primary analyses focused on within-group pre- and post-intervention comparisons in the HIRT group.

## Results

The baseline characteristics of the participants were comparable between the HIRT and CG groups and presented in Table 1, with no statistically significant differences observed across the measured variables (*p* > 0.05). Handgrip strength measurements for both the right and left hands were slightly lower in the HIRT group; however, the differences were not statistically significant. Cardiometabolic parameters were similarly aligned between the groups, ensuring homogeneity at baseline. The effect sizes (Cohen's *d*) for all comparisons ranged from 0.01 to 0.18, indicating negligible to small effects. All statistically significant improvements were observed exclusively in the HIRT group. The control group did not show significant changes in any of the outcomes assessed.

**Table 1:** Baseline characteristics of older participants enrolled in the study

	CG	HIRT	*p*-value/effect size (*d*)
	M ± SD		
*Biosocial*			
Gender (n, F/M)	17/6	18/5	–
Age (years)	66.71 ± 4.98	66.91 ± 5.26	0.87/*d* = 0.04
Height (cm)	161 ± 6	159 ± 7	0.50/*d* = 0.30
Weight (kg)	69.43 ± 9.74	70.45 ± 10.75	0.75/*d* = 0.10
BMI (kg/m^2^)	28.01 ± 3.66	27.98 ± 3.97	0.97/*d* = 0.01
*Physical fitness e body composition*			
Body Fat (%)	38.40 ± 7.18	39.75 ± 7.03	0.53/*d* = 0.19
Body Lean (%)	61.49 ± 7.02	60.14 ± 6.82	0.53/*d* = 0.19
Hand Strength Right (kg)	28.05 ± 6.95	27.32 ± 7.01	0.73/*d* = 0.10
Hand Strength Left (kg)	27.34 ± 5.27	25.23 ± 7.58	0.38/*d* = 0.33
*Cardiometabolic*			
Systolic Blood Pressure (mmHg)	136.54 ± 16.63	135.22 ± 18.33	0.84/*d* = 0.07
Diastolic Blood Pressure (mmHg)	78.04 ± 9.23	76.17 ± 11.04	0.64/*d* = 0.18
Pulse Pressure (mmHg)	58.21 ± 12.73	59.04 ± 13.79	0.84/*d* = 0.06
Heart Rate (bpm)	81.24 ± 12.02	82.83 ± 11.06	0.72/*d* = 0.14
Maximal Inspiratory Pressure (cm H₂O)	−76.48 ± 27.48	−79.17 ± 31.64	0.79/*d* = 0.09
Maximal Expiratory Pressure (cm H₂O)	83.14 ± 21.43	82.83 ± 23.20	0.96/*d* = 0.01

M ± SD, mean and standard deviation; n, sample size; F, female; M, male; BMI, body mass index; %, percentage; cm, centimeters; kg/m^2^, kilograms per square meter; mmHg, millimeters of mercury; bpm, beats per minute; cm H₂O, centimeters of water; *p*-values reflect significance testing between CG (control group) and HIRT (high intensity resistance training) at baseline; Data on gender are expressed as counts (n) and were not analyzed statistically

### Effects on pulmonary (breath condensate) cytokines

**Fig. 1:**
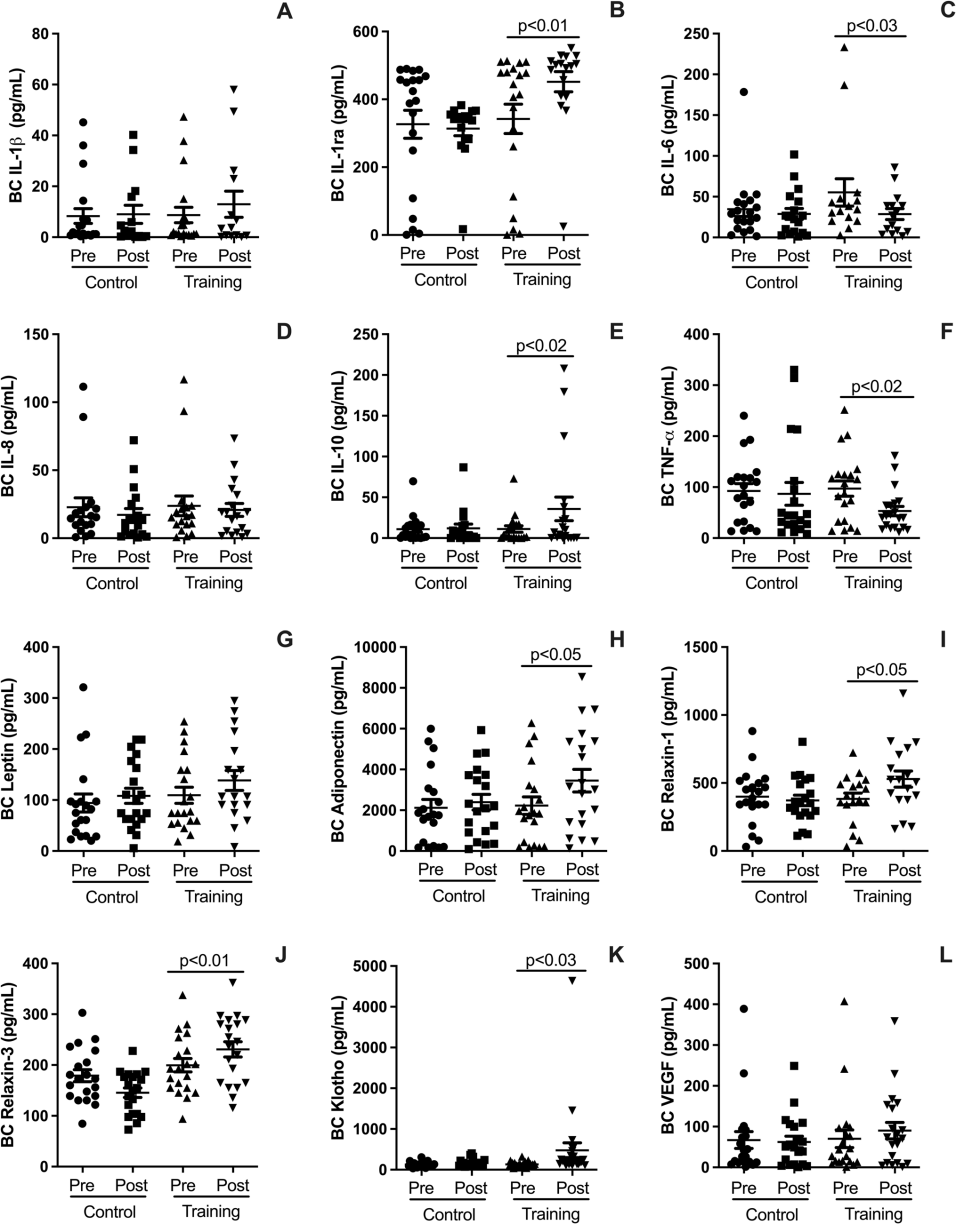
HYPERLINK "sps:id::fig1||locator::gr1||MediaObject::0" Plots illustrate pre- and post-intervention differences for the HIRT and CG groups in pulmonary cytokine levels measured via breath condensate. These include the anti-inflammatory cytokines IL-1ra (**B**), IL-10 (**E**), and Klotho (**K**), as well as Relaxin-1 (**I**) and Relaxin-3 (**J**). Pro-inflammatory cytokines measured include IL-1β (**A**), IL-8 (**D**), IL-6 (**C**), TNF-α (**F**), and Leptin (**G**), along with VEGF (**L**). Data are expressed as mean ± standard deviation. Statistical significance was determined using a paired t-test, with *p* < 0.05 considered significant

The results illustrated in Fig. 1 reveal several significant effects of HIRT on pulmonary cytokines measured via breath condensate. HIRT significantly increased the levels of anti-inflammatory cytokines, including IL-1ra (Fig. 1B; *p* < 0.02) and IL-10 (Fig. 1E; *p* < 0.05). Additionally, HIRT was associated with elevated levels of Klotho (Fig. 1K; *p* < 0.04), a protein known for its anti-inflammatory, anti-fibrotic, and protective properties. Similarly, the levels of Relaxin-1 (Fig. 1I; *p* < 0.05) and Relaxin-3 (Fig. 1J; *p* < 0.01), both of which exhibit anti-inflammatory and anti-fibrotic effects, were significantly increased.

On the other hand, no significant changes were observed in the pulmonary levels of adiponectin (Fig. 1H; *p* > 0.05) or pro-inflammatory cytokines IL-1β (Fig. 1A; *p* > 0.05) and IL-8 (Fig. 1D; *p* > 0.05). However, HIRT led to a notable reduction in other pro-inflammatory markers, including IL-6 (Fig. 1C; p < 0.04), TNF-α (Fig. 1F; *p* < 0.04), and leptin (Fig. 1G; *p* < 0.05). Additionally, HIRT significantly increased the levels of the growth factor VEGF (Fig. 1L; *p* < 0.05), which plays a key role in pulmonary repair and vascular regeneration.

### Effects on serum cytokines

**Fig. 2:**
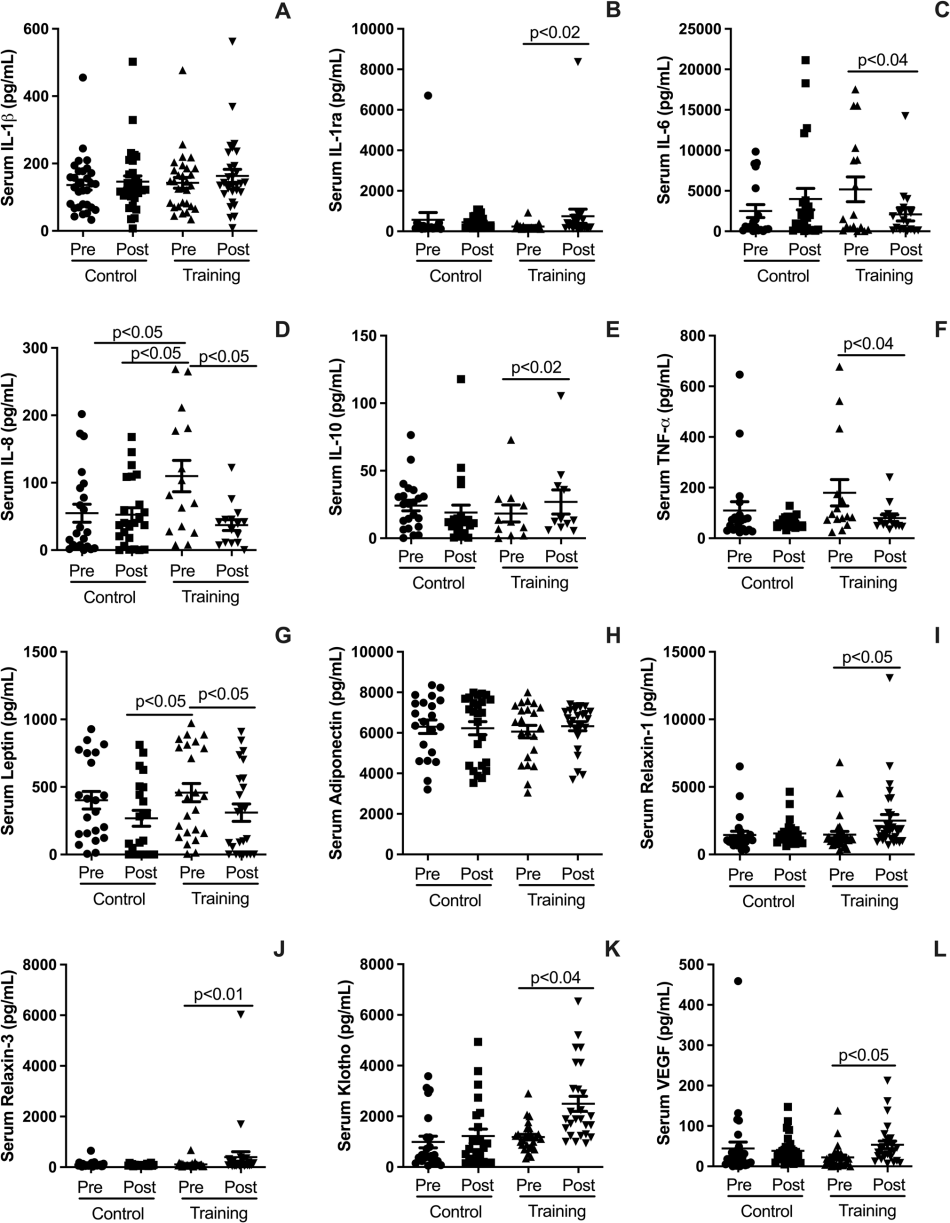
Plots illustrate pre- and post-intervention differences for the HIRT and CG groups in serum cytokine levels. These include the anti-inflammatory cytokines IL-1ra (**B**), IL-10 (**E**), and Klotho (**K**), as well as Relaxin-1 (**I**) and Relaxin-3 (**J**). Pro-inflammatory cytokines measured include IL-1β (**A**), IL-8 (**D**), IL-6 (**C**), TNF-α (**F**), and leptin (**G**). Additionally, VEGF levels (**L**) were evaluated. Data are expressed as mean ± standard deviation. Statistical significance was determined using a paired t-test, with *p* < 0.05 considered significant

Figure 2 illustrates the impact of HIRT on serum cytokines, highlighting both anti-inflammatory and pro-inflammatory responses. HIRT significantly increased serum levels of anti-inflammatory cytokines IL-1ra (Fig. 2B; *p* < 0.02) and IL-10 (Fig. 2E; *p* < 0.05). Furthermore, it elevated the levels of Klotho (Fig. 2K; *p* < 0.04), a protein associated with anti-inflammatory, anti-fibrotic, and anti-aging properties, as well as Relaxin-1 (Fig. 2I; *p* < 0.05) and Relaxin-3 (Fig. 2J; *p* < 0.01), both known for their anti-inflammatory and anti-fibrotic roles.

Interestingly, HIRT did not affect adiponectin levels (Fig. 2H; *p* > 0.05) or pro-inflammatory cytokines IL-1β (Fig. 2A; *p* > 0.05) and IL-8 (Fig. 2D; *p* > 0.05). However, it significantly reduced other pro-inflammatory markers, including IL-6 (Fig. 2C; *p* < 0.04), TNF-α (Fig. 2F; *p* < 0.04), and leptin (Fig. 2G; *p* < 0.05). Additionally, HIRT increased the levels of the growth factor VEGF (Fig. 2L; *p* < 0.05), which is linked to vascular health and tissue regeneration.

### Effects on blood and pulmonary leukocytes

**Fig. 3:**
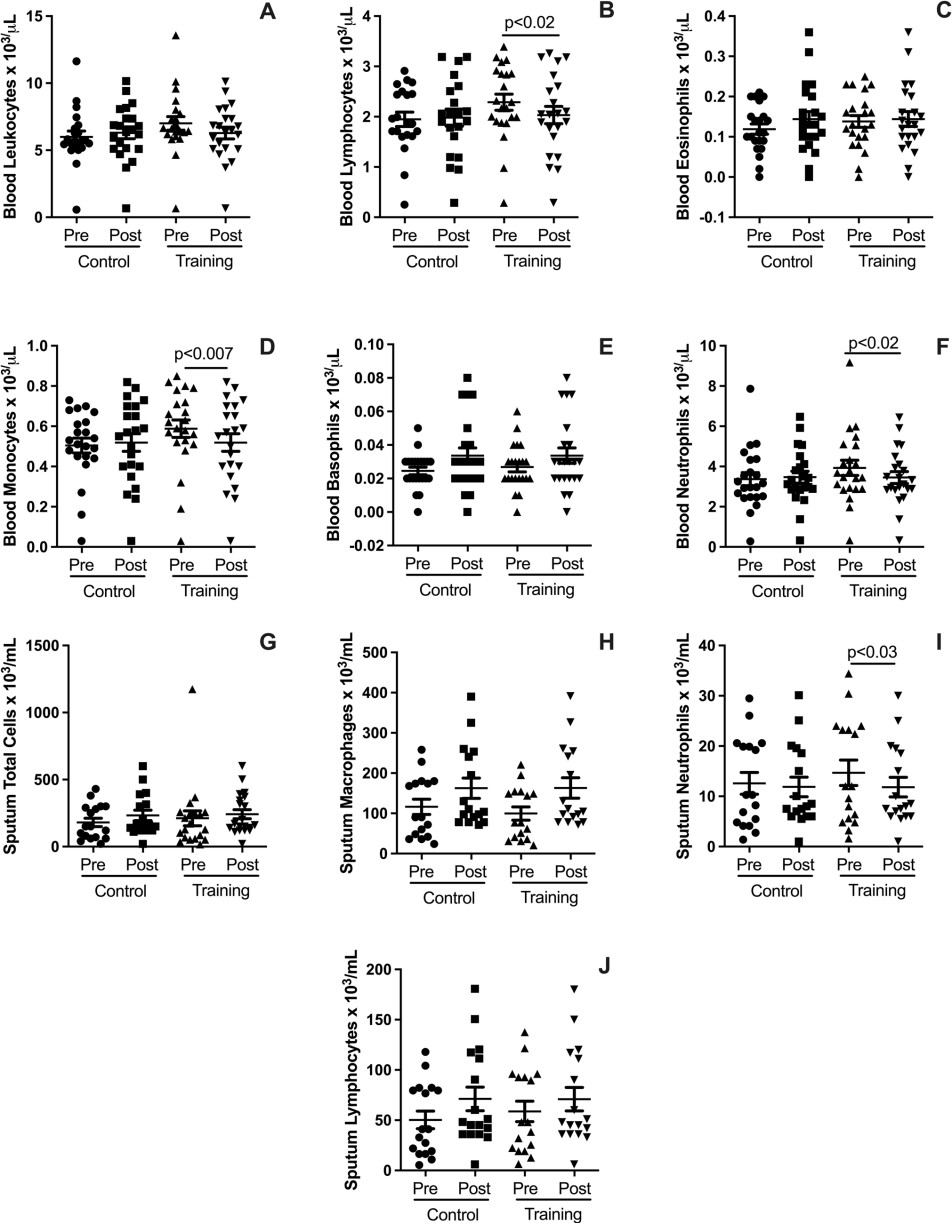
Plots illustrate pre- and post-intervention differences for the HIRT and CG groups in leukocyte populations from blood and pulmonary samples. Blood outcomes include total leukocyte counts (**A**), lymphocyte counts (**B**), eosinophil counts (**C**), monocyte counts (**D**), basophil counts (**E**), and neutrophil counts (**F**). Pulmonary outcomes (induced sputum) include total leukocyte counts (**G**), macrophage counts (**H**), neutrophil counts (**I**), and lymphocyte counts (**J**). Data are expressed as mean ± standard deviation. Statistical significance was determined using a paired t-test, with p < 0.05 considered significant

**Fig. 4:**
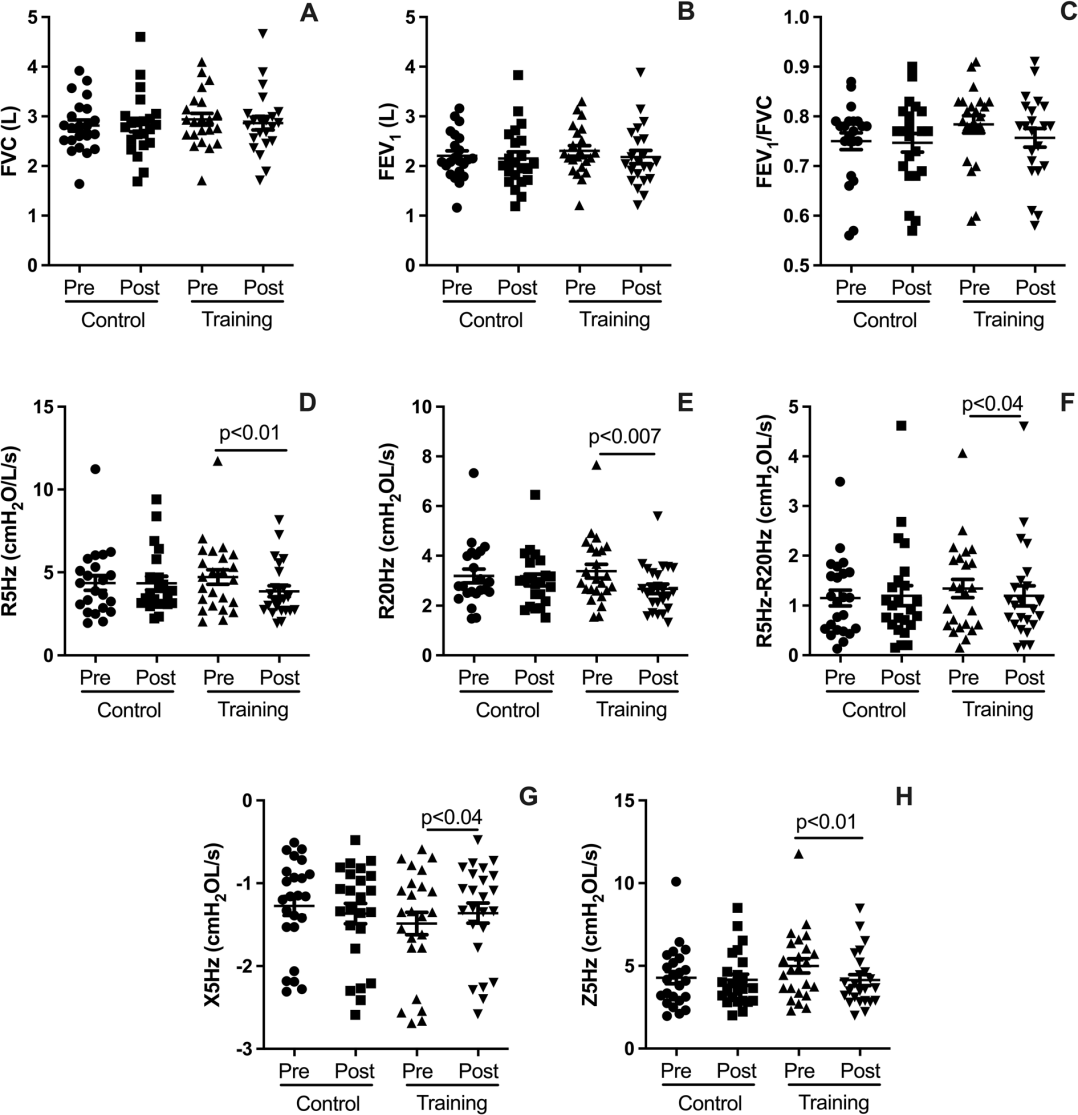
Plots illustrate pre- and post-intervention differences for the HIRT and CG groups in lung function and mechanics. Spirometry outcomes include forced vital capacity (FVC, **A**), forced expiratory volume in one second (FEV₁, **B**), and the FEV₁/FVC ratio (**C**). Lung mechanics outcomes include total respiratory resistance (R5Hz, **D**), proximal airway resistance (R20Hz, **E**), distal airway resistance (R5–R20Hz, **F**), respiratory system reactance (X5Hz, **G**), and respiratory system impedance (Z5Hz, **H**). All measurements are reported in cmH₂O·L⁻^1^·s⁻^1^. Data are expressed as mean ± standard deviation. Statistical significance was determined using a paired t-test, with *p* < 0.05 considered significant

Figure 3 highlights the effects of HIRT on leukocyte populations in blood and pulmonary samples. In the blood, HIRT significantly reduced lymphocyte counts (Fig. 3B; *p* < 0.05), monocyte counts (Fig. 3D; *p* < 0.007), and neutrophil counts (Fig. 3F; *p* < 0.02). However, no significant changes were observed in total leukocyte counts (Fig. 4A), eosinophil counts (Fig. 3C), or basophil counts (Fig. 3E). In the lungs (analyzed via induced sputum), HIRT led to a significant decrease in neutrophil counts (Fig. 3I; *p* < 0.03). Conversely, it did not significantly affect total pulmonary leukocyte counts (Fig. 3G), macrophage counts (Fig. 3H), or lymphocyte counts (Fig. 3J).

### Effects on lung function and mechanics

Figure 4 presents the effects of HIRT on lung function and mechanics after five weeks of intervention. Regarding spirometry outcomes, HIRT did not produce significant changes in forced vital capacity (FVC, Fig. 4A; *p* > 0.05), forced expiratory volume in one second (FEV_1_, Fig. 4B; *p* > 0.05), or the FEV_1_/FVC ratio (Fig. 4C; *p* > 0.05). However, HIRT significantly improved lung mechanics, as shown by reductions in total respiratory system resistance (R5Hz, Fig. 4D; *p* < 0.01), proximal airway resistance (R20Hz, Fig. 4E; *p* < 0.007), and distal airway resistance (R5-R20Hz, Fig. 4F; *p* < 0.04). Additionally, HIRT reduced respiratory system reactance (X5Hz, Fig. 4G; *p* < 0.04) and respiratory system impedance (Z5Hz, Fig. 4H; *p* < 0.01).

### Effects of HRTI on PFF and HrQoL

**Fig. 5:**
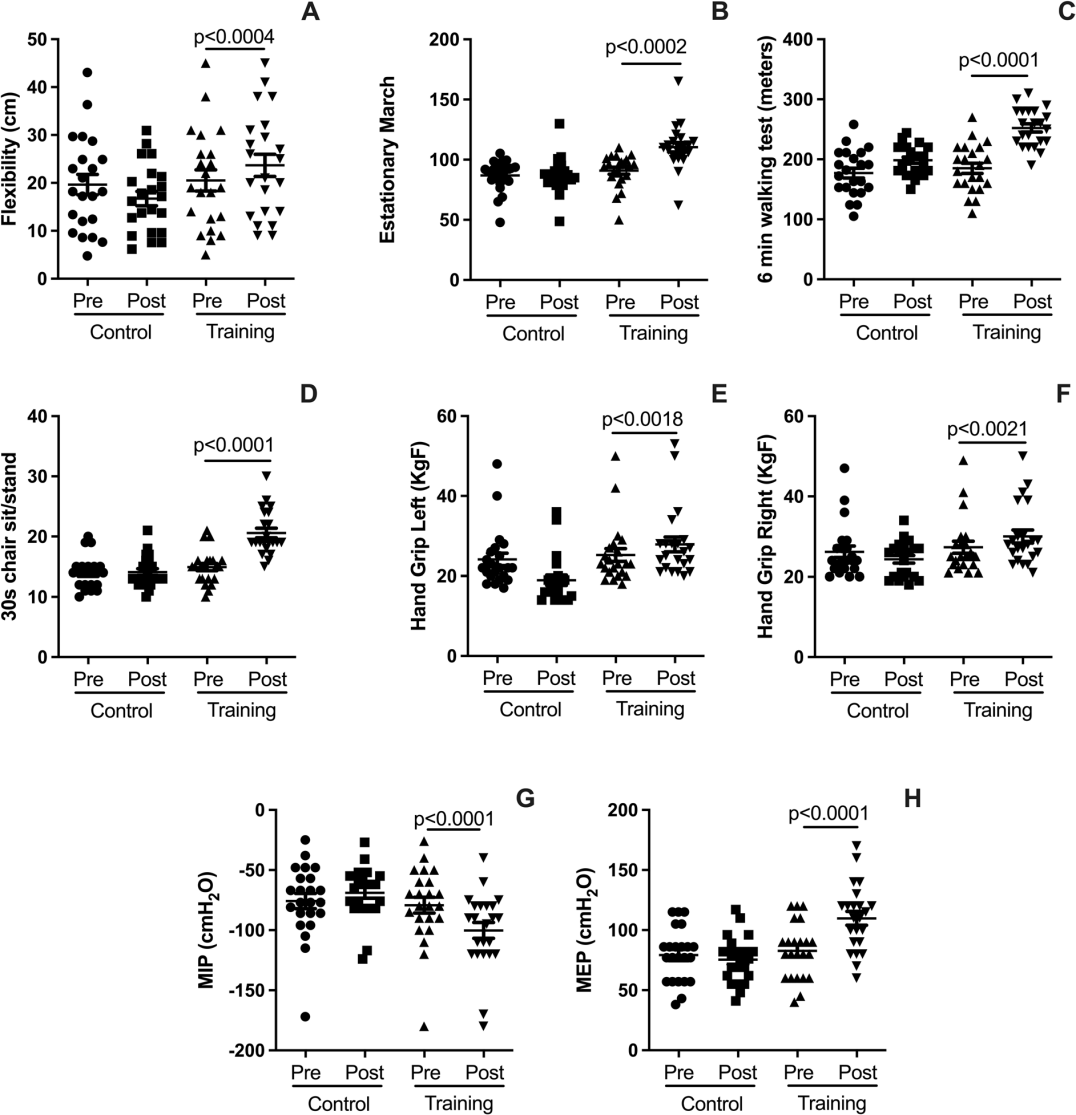
Plots illustrate pre- and post-intervention differences for the HIRT and CG groups in flexibility (**A**), performance in the stationary march test (SMT; **B**), distance covered in the 6-min walk test (6MWT; **C**), anaerobic capacity measured by the 30-s sit-to-stand test (30 s-SS; **D**), left-hand grip strength (**E**), right-hand grip strength (**F**), maximal inspiratory pressure (MIP; **G**), and maximal expiratory pressure (MEP; **H**). Data are expressed as mean ± standard deviation. Statistical significance was determined using a paired t-test, with *p* < 0.05 considered significant

Figure 5 highlights the significant improvements in PFF achieved through HIRT. After the intervention, participants exhibited enhanced flexibility (Fig. 5A; *p* < 0.0004), improved performance in the SMT (Fig. 5B; *p* < 0.0002), and increased distance covered during the 6MWT (Fig. 5C; *p* < 0.0001). Additionally, anaerobic capacity, assessed through the 30 s-SS test, showed marked improvement (Fig. 5D; *p* < 0.0001). Handgrip strength increased significantly in both the left (Fig. 5E; *p* < 0.0018) and right hands (Fig. 5F; *p* < 0.0021). Furthermore, respiratory muscle strength improved substantially, with higher MIP (Fig. 5G; *p* < 0.0001) and MEP (Fig. 5H; *p* < 0.0001). Additionally, the different subdimensions of the HrQoL scales exhibited statistically significant improvements after the HIRT intervention, whereas the CG showed no improvements (see Supplementary Fig. S1; *p* < 0.05). Further details on initial and post-intervention maximal strength values for each exercise are provided in Supplementary Table S1.

### Effects on body composition and anthropometrics

**Fig. 6:**
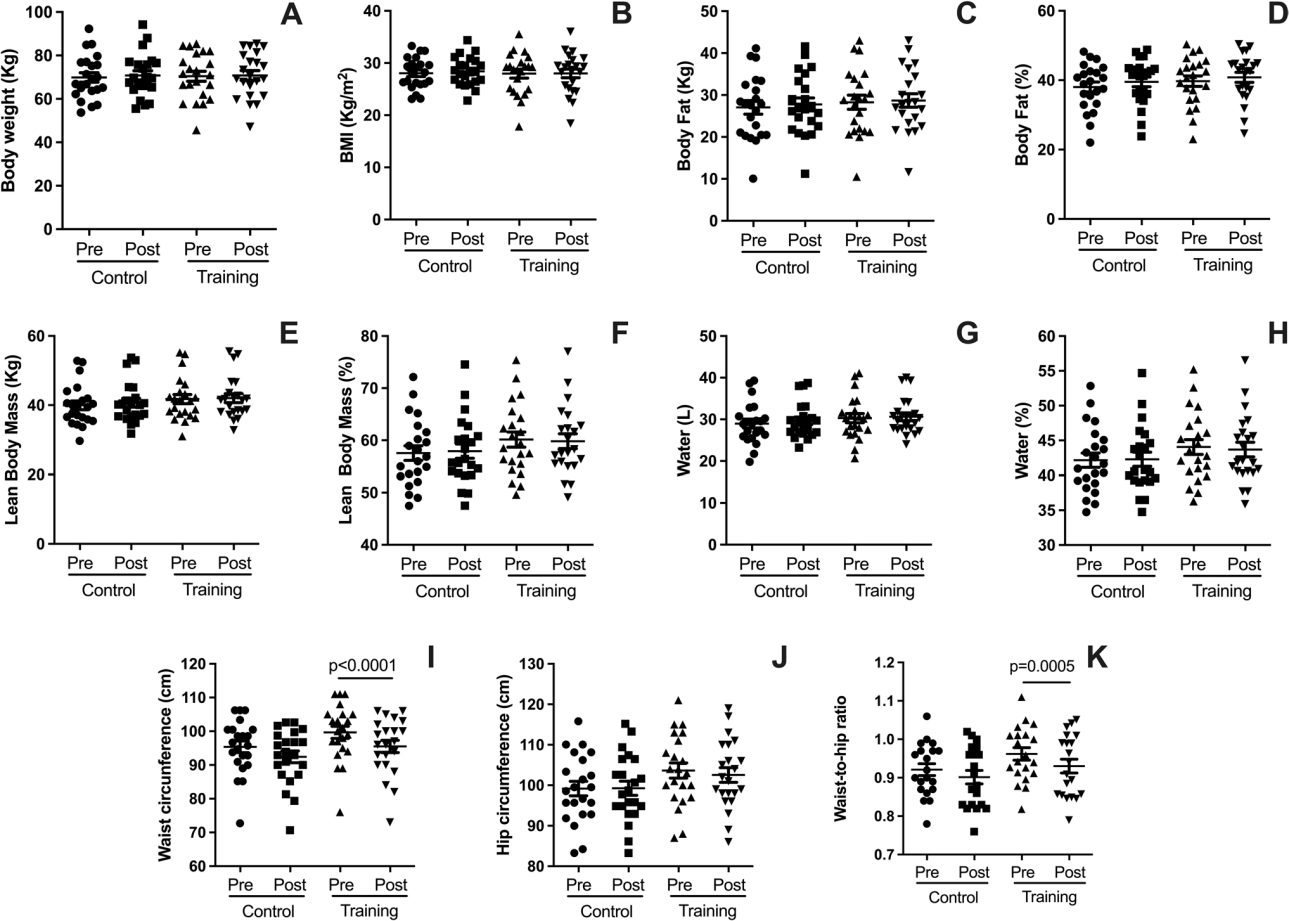
Plots illustrate pre- and post-intervention differences for the HIRT and CG groups in body composition and anthropometric variables: body weight (**A**), body mass index (BMI; **B**), body fat mass (kg; **C**), body fat percentage (**D**), lean body mass (kg; **E**), lean body mass percentage (**F**), total body water (liters; **G**), body water percentage (Fig. 6H), hip circumference (**J**), and waist-to-hip ratio (**K**). Data are expressed as mean ± standard deviation. Statistical significance was determined using a paired t-test, with *p* < 0.05 considered significant

The impact of HIRT on body composition and anthropometric measurements is presented in Fig. 6. Following five weeks of intervention, no significant changes were observed in all variables. However, significant reductions were recorded in waist circumference (Fig. 6I; *p* < 0.0001) and the waist-to-hip ratio (Fig. 6K; *p* < 0.0005).

## Discussion

The present study is the first to demonstrate that five weeks of HIRT trigger a pronounced anti-inflammatory and anti-fibrotic response at both systemic and pulmonary levels, effectively mitigating the pro-inflammatory effects commonly associated with metabolic syndrome in older adults. Furthermore, HIRT strengthened lung mechanics and respiratory muscle function—critical protective factors that may contribute to better outcomes during hospitalization. Notably, HIRT significantly improved functional physical capacity, reduced pain, and enhanced overall HrQoL, independent of reductions in body fat. These favorable outcomes suggest that HIRT may be an effective intervention for improving health in older adults with MetS. This is particularly relevant in light of recent epidemiological data from inland Portugal, where older populations exhibit high rates of hypertension, dyslipidemia, and overweight/obesity—hallmarks of MetS—within contexts of limited healthcare access (Lindo et al. 2024). The lack of significant changes in the control group further supports that the observed improvements were attributable to the HIRT intervention rather than spontaneous variations over time.

### HIRT effects on body composition

The HIRT regimen did not result in significant changes in overall body composition, apart from reductions in waist circumference and waist-to-hip ratio, indicating localized improvements in central adiposity. These findings may be attributed to the short duration of the intervention, as medium- to long-term studies tend to yield more pronounced effects on body composition. Longer interventions have been shown to promote significant reductions in body fat and improvements in lean mass due to the cumulative effects of HIRT on metabolism and muscle hypertrophy (Currier et al. 2023). While the current study specifically investigated the effects of HIRT and not HIIT (high intense interval training) (D’Amuri et al. 2021), it is noteworthy that the observed reductions in waist circumference and waist-to-hip ratio represent a meaningful outcome. Increased waist circumference and waist-to-hip ratio are well-established risk factors for cardiovascular diseases (Huxley et al. 2010), and their significant reduction in the present study highlights the potential of HIRT to mitigate cardiovascular risk factors in older adults with MetS.

### HIRT effects on PFF and HrQoL

HIRT promoted rapid and significant improvements in several PFF status, including increased flexibility, better performance in muscle-strength tests, enhanced walkability, and notably, improved maximal inspiratory and expiratory pressures, indicating stronger respiratory muscles. Respiratory muscle strength plays a key role in recovery during hospitalization and mechanical ventilation, particularly in cases of severe pneumonia, where it is a major factor in successful weaning from mechanical support (Cader et al. 2010; Réginault et al. 2024). The rapid gains in PFF observed in older adults with MetS can be attributed to the structured nature of the HIRT protocol, which targets key functional domains. Additionally, the low baseline PFF levels of the participants likely provided a substantial opportunity for improvement, allowing even short-term interventions to yield significant results. These findings align with previous research on older adults using moderate to high-intensity resistance training (Marcos-Pardo et al. 2019). Thus, the broad functional benefits gained from HIRT strongly support its application in older adults with MetS.

HIRT showed a positive impact on all subdimensions of HrQoL scale (Supplementary Fig. S1). The improvement in HrQoL, despite the short intervention period, may be attributed to enhanced lung function and improved PFF status, contributing to better overall well-being and reduced discomfort. These findings are consistent with previous studies demonstrating the safety and efficacy of moderate-intensity resistance training in improving HrQoL among healthy older adults (Pedersen et al. 2017; Hart and Buck 2019). Additionally, similar benefits have been observed in cancer survivors aged 25–70 years, further supporting the positive effects of HIRT on HrQoL in diverse populations (Backer et al. 2008; Beebe-Dimmer et al. 2023).

### Effects on lung function and mechanics

While HIRT did not significantly impact key spirometry measurements such as FVC, FEV_1_, or the FEV_1_/FVC ratio, it notably improved lung mechanics. Specifically, HIRT led to reductions in total respiratory system resistance (R5Hz), as well as resistance in both the proximal (R20Hz) and distal (R5–R20Hz) airways. Furthermore, HIRT positively influenced pulmonary impedance (Z5Hz) and reactance (X5Hz), both of which reflect distal airway mechanics. These findings are particularly significant, as the impulse oscillometry system (IOS) is highly sensitive in detecting subtle pulmonary changes, including those associated with obesity. This is the first study to demonstrate that HIRT improves all parameters of lung mechanics, which are known to be negatively affected by both aging and MetS (Brandao-Rangel et al. 2021). The observed improvements may reflect beneficial effects on lung remodeling, although additional imaging methods, such as computed tomography, are necessary for a more comprehensive assessment.

### Effects on blood and pulmonary leukocytes

The impact of HIRT on blood and pulmonary leukocyte populations was also evaluated. HIRT significantly reduced circulating lymphocytes, monocytes, and neutrophils, as well as pulmonary neutrophils, suggesting both systemic and pulmonary anti-inflammatory effects. These findings are particularly important considering the low-grade systemic inflammation characteristic of MetS, which is associated with elevated inflammatory cell counts. Chronic inflammation plays a central role in the development of atherogenesis, dyslipidemia, type 2 diabetes, and systemic arterial hypertension, all of which are common features of MetS (Andersen et al. 2016; Zafar et al. 2018; Brandao-Rangel et al. 2021). The rapid changes observed in blood and pulmonary leukocytes following a short-term protocol may be attributed to the acute inflammatory response induced by HIRT, which triggers the release of anti-inflammatory cytokines and mobilization of leukocytes into systemic circulation and pulmonary sites (Burini et al. 2020). This response is thought to be a transient adaptation to physical stress, with subsequent reductions in inflammatory markers driven by the anti-inflammatory effects of regular exercise.

### Effects on serum cytokines

The HIRT protocol produced significant effects on serum cytokine levels, particularly by increasing the anti-inflammatory cytokines IL-1ra and IL-10, which are known to protect against inflammation and promote improved lung function. Additionally, HIRT elevated the levels of Klotho, a protein associated with anti-inflammatory and anti-fibrotic properties, as well as Relaxin-1 and Relaxin-3, both of which also exert anti-inflammatory and anti-fibrotic effects. The observed reductions in blood and pulmonary leukocytes following this short-term HIRT protocol may be explained by exercise-induced modulation of the immune response, whereby acute bouts of resistance training promote a shift toward an anti-inflammatory profile, reducing the number of circulating inflammatory cells (Santiago et al. 2018). These findings indicate that HIRT induces systemic and pulmonary increases in key proteins, offering new insights into the potential mechanisms through which HIRT may mitigate the inflammatory and fibrotic effects associated with MetS (Heringlake et al. 2009; Santos-Dias et al. 2017).

### Effects on pulmonary (breath condensate) cytokines

HIRT also demonstrated a significant impact on pulmonary cytokines, measured via breath condensate. Specifically, HIRT increased the levels of anti-inflammatory cytokines such as IL-1ra and IL-10, as well as anti-fibrotic proteins like Klotho, Relaxin-1, and Relaxin-3 within the pulmonary environment. In contrast, no significant changes were observed in pulmonary levels of adiponectin or pro-inflammatory cytokines such as IL-1β and IL-8. The elevation of these anti-inflammatory and anti-fibrotic markers in both blood and lungs further supports the potential benefits of HIRT in managing inflammation and fibrosis, particularly in the context of MetS (Heringlake et al. 2009; Andersen et al. 2016). The significant increase in anti-inflammatory and anti-fibrotic cytokines in pulmonary breath condensate following HIRT suggests that this protocol may modulate local lung inflammation and fibrosis, potentially improving pulmonary function by enhancing the balance between pro- and anti-inflammatory mediators (Araneda et al. 2016; Uzeloto et al. 2022).

### Strengths and limitations

This study offers several strengths, including the use of a well-established HIRT protocol in older adults with MetS, which enabled the evaluation of both pulmonary and systemic outcomes. Additionally, the use of advanced techniques such as impulse oscillometry for assessing lung mechanics and serum cytokine analysis provided valuable insights into the effects of HIRT on inflammation and pulmonary function.

However, the study also presents some notable limitations. The short duration of the intervention and the relatively small sample size limit the generalizability of the findings, although these constraints are inherent to the nature of a feasibility study. Furthermore, the study did not include a direct assessment of lung remodeling or a long-term follow-up, both of which are crucial for fully understanding the sustainability of the observed benefits.

### Practical applications in the real world

The findings from this study have important practical applications for real-world settings. The short-term HIRT protocol, requiring only twice-weekly sessions, can be readily incorporated into existing healthcare routines for older adults with MetS, offering a time-efficient and effective intervention. Given that HIRT enhances physical-functional capacity, muscle strength, and immune responses, it may serve as a valuable non-pharmacological strategy for improving the health outcomes in this population. This approach may be particularly beneficial for individuals who are unable or unwilling to engage in longer or more intensive exercise regimens. However, although the HIRT protocol was well tolerated in this study, careful screening, supervision, and individualized progression are essential to minimize the potential risk of musculoskeletal injuries in older adults.

### Future perspectives on pulmonary rehabilitation

Future research on pulmonary rehabilitation could build upon the promising effects of HIRT demonstrated in this study. Investigating the long-term impacts of HIRT on lung function, lung remodeling, and overall health outcomes in older adults with MetS is essential. Additionally, exploring the combination of HIRT with other pulmonary rehabilitation strategies, such as aerobic training and breathing exercises, could provide valuable insights into optimizing respiratory health in this population. Advanced imaging techniques, such as computed tomography, should also be incorporated in future studies to assess structural changes in the lungs and to evaluate the potential for enhanced lung remodeling.

## Conclusion

The five-week, short-term HIRT protocol, conducted twice per week, demonstrated remarkable efficacy in enhancing lung mechanics and bolstering systemic and pulmonary immune responses, including the promotion of anti-inflammatory and anti-fibrotic processes. Although it did not produce significant changes in pulmonary function, HIRT significantly improved respiratory and peripheral muscle strength, as well as functional capacity. These findings highlight the potential of non-pharmacological interventions to improve the health of older adults with MetS, offering a promising strategy for enhancing both quality of life and physical fitness in this population.

## Supplementary Information

Below is the link to the electronic supplementary material.

Supplementary file1 (TIFF 3100 KB)

Supplementary file2 (DOCX 16 KB)

## Data Availability

All raw data will be available upon a request to the corresponding author, under a reasonable reason.
