# Supplementary material for: Short-term high-intensity resistance training: a feasibility study on pulmonary, immune and physical-functional fitness benefits for older adults with metabolic syndrome
Source: Eur J Appl Physiol. 2025 Jul 26;126(1):437–53. doi: 10.1007/s00421-025-05920-0 (PMC12881092; doi:10.1007/s00421-025-05920-0)
Supplement: Supplementary file 2 — Supplementary file2 (DOCX 16 KB) [file 421_2025_5920_MOESM2_ESM.docx]

| **Exercise** | **Men (n=5)** | | **Woman (n=18)** | |
| --- | --- | --- | --- | --- |
|  | **Initial 1 RM**  **(kg)** | **Final 1RM**  **(kg)** | **Initial 1 RM**  **(kg)** | **Final 1RM**  **(kg)** |
| Bent-over barbell row | 13 ± 1 | 16 ± 1 | 12 ± 2 | 15 ± 2 |
| Deadlift | 14 ± 1 | 18 ± 1 | 13 ± 2.5 | 17 ± 2 |
| Flat bench press | 20 ± 2 | 21 ± 2 | 18 ± 4 | 20 ± 4 |
| 45° leg press | 53 ± 4 | 75 ± 6 | 51 ± 7 | 73 ± 10 |
| ***Notes:*** Data are presented as mean ± standard deviation; n = sample size; kg = kilograms. | | | | |

Table S1: Initial and final one-repetition maximum (1RM) values for each exercise, separated by sex.
